# An outline of necrosome triggers

**DOI:** 10.1007/s00018-016-2189-y

**Published:** 2016-04-06

**Authors:** Tom Vanden Berghe, Behrouz Hassannia, Peter Vandenabeele

**Affiliations:** 1 Inflammation Research Center, VIB, 9000 Ghent, Belgium; 2 Department of Biomedical Molecular Biology, Ghent University, 9000 Ghent, Belgium; 3 Laboratory of Eukaryotic Gene Expression and Signal Transduction, Department of Physiology, Ghent University, 9000 Ghent, Belgium

**Keywords:** Necroptosis, RIPK1, RIPK3, MLKL, RHIM, Pathogens

## Abstract

Necroptosis was initially identified as a backup cell death program when apoptosis is blocked. However, it is now recognized as a cellular defense mechanism against infections and is presumed to be a detrimental factor in several pathologies driven by cell death. Necroptosis is a prototypic form of regulated necrosis that depends on activation of the necrosome, which is a protein complex in which receptor interacting protein kinase (RIPK) 3 is activated. The RIP homotypic interaction motif (RHIM) is the core domain that regulates activation of the necrosome. To date, three RHIM-containing proteins have been reported to activate the kinase activity of RIPK3 within the necrosome: RIPK1, Toll/IL-1 receptor domain-containing adaptor inducing IFN-β (TRIF), and DNA-dependent activator of interferon regulatory factors (DAI). Here, we review and discuss commonalities and differences of the increasing number of activators of the necrosome. Since the discovery that activation of mixed lineage kinase domain-like (MLKL) by RIPK3 kinase activity is crucial in necroptosis, interest has increased in monitoring and therapeutically targeting their activation. The availability of new phospho-specific antibodies, pharmacologic inhibitors, and transgenic models will allow us to further document the role of necroptosis in degenerative, inflammatory and infectious diseases.

## Introduction

Rudolf Virchow (1821–1902, Prussia), founder of the cell theory (*Omnis cellula e cellula*) and cellular pathology, referred to tissue injury as “parenchymatous inflammation” and introduced the idea that tissue injury is caused by pathological changes within the cells. In 1858, he introduced the notion of cell death as a potential basis for pathology, with ‘necrobiosis’ being a physiological process of spontaneous wearing out of living parts from the body and ‘necrosis’ an accidental process. The term ‘necrosis’ comes from the Greek word ‘nekros,’ which means ‘dead body.’ Virchow’s necrobiosis–necrosis dichotomy resembles to some extent the current apoptosis–necrosis classification [1]. Together with cellular and molecular insights into inflammation came a shift in our understanding of the molecular interplay between cell death and inflammation at the site of tissue injury. This emerging field of research is crucial for understanding organismal homeostasis and how its processes contribute to a growing list of inflammatory and degenerative pathologies. Although cell death during inflammation was initially considered a manifestation of tissue damage, it was later recognized as a mechanism for eliminating pathogens and regulating inflammation by exposing or releasing molecular patterns that attract and alter the functions of other cells [2]. More recently, it became clear that phagocytosis of apoptotic cells can also initiate anti-inflammatory and tissue-regenerative responses [3, 4].

The current notion that not only apoptotic but also necrotic cell death is molecularly controlled by defined signaling mechanisms has increased the interest in studying regulated necrosis in the context of development, homeostasis and inflammation. Although RIPK3 knockout mice and MLKL knockout mice do not show an overt phenotypic abnormality under non-challenged conditions [5–8], it became clear that RIPK3-mediated necroptosis is a highly controlled cell death program that is executed when negative regulators such as caspase-8, IAPs or even RIPK1 are absent [9–17]. This suggests that the default mode during development and homeostasis is strong inhibition of the necroptosis pathway. Regulated necrosis, like passive necrosis due to physico-chemical insult, is caused by loss of plasma membrane integrity leading to cellular rounding followed by swelling (oncosis). When the immune system recognizes cellular content exposed or released due to loss of membrane integrity, it initiates an inflammatory response. Regulated necrosis can be classified into several cell death modalities, such as necroptosis, parthanatos, ferroptosis, cyclophilin D-dependent necrosis, (n)etosis and pyroptosis [18]. Each type of regulated necrosis has particular biochemical features, yet it is not clear whether the common morphological features of these forms of cell death share or converge on common pathways. Other forms of cellular death are being identified, such as entosis [19], autosis [20, 21] and autoschizis [22].

Necroptosis, the best-characterized form of regulated necrosis, is mediated by the concerted action of receptor interacting protein kinase (RIPK) 3 and mixed lineage kinase domain-like (MLKL). In this review, we provide a snapshot of the activation of RIPK3 within the necrosome, typically by the three RHIM-containing proteins RIPK1, Toll/IL-1 receptor domain-containing adaptor inducing IFN-β (TRIF) and DNA-dependent activator of interferon regulatory factors (DAI). We briefly discuss the pluripotent roles of RIPK1 and RIPK3 in gene regulation and cell death induction.

## RIPK1-dependent necroptosis

The mechanism of RIPK1-dependent necroptosis has been discovered mostly from studying tumor necrosis factor (TNF) signaling under conditions favoring cell death. This is because the major function of TNFR1, like that of DR3 (TRAMP/APO-3), is to induce pro-survival and pro-inflammatory genes, in contrast to some other death receptor family members such as CD95 (FAS/APO-1), TRAILR1 (DR4) and TRAILR2 (APO-2/TRICK/DR5/KILLER). The following model is currently proposed for TNFR1 signaling: sensing of trimeric TNF by TNFR1 induces the assembly of a primary receptor-bound complex that triggers activation of signaling pathways leading to gene induction [23–25]. Subsequently, assembly of a secondary TNFR1-unbound cytosolic complex induces cell death. For FAS and TRAILR1/2, the opposite situation is observed [26]. This sequential signaling provides a backup response by the secondary cytosolic complex in case the default pathway activated by the receptor-associated complex fails to resolve the infectious or inflammatory condition. Typically, pathogens and (epi)genetic factors can interfere with gene activation or cell death induction. Thus, this sequential signaling probably evolved as a host defense against pathogens or conditions that might perturb either pathway.

**Fig. 1:**
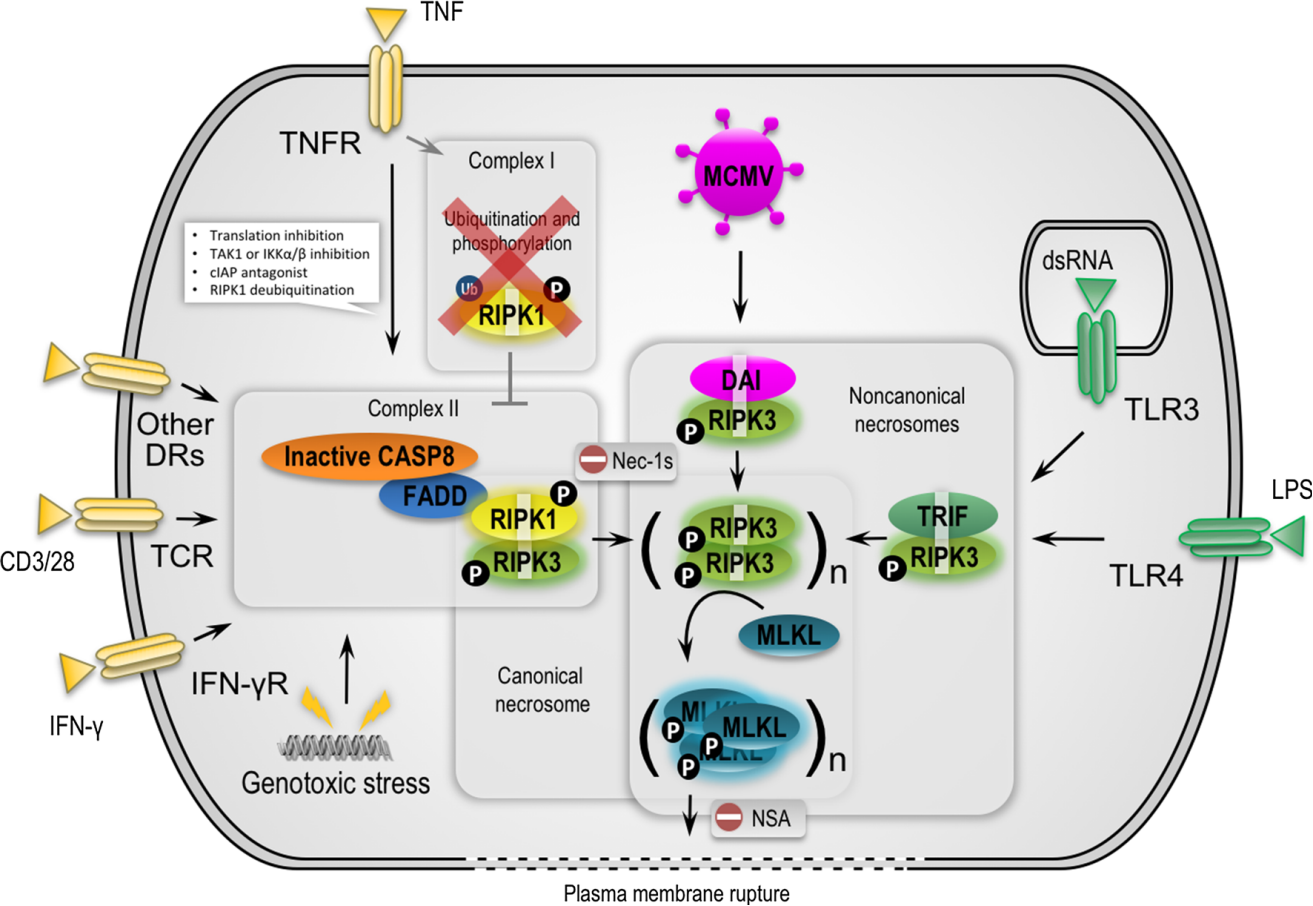
Canonical versus noncanonical necrosome activation by three distinct RHIM-containing host adaptors. RIPK1 is a key pro-necrotic kinase that responds to several DRs, TCR, IFN-γR and genotoxic stress by forming a RIPK1–RIPK3 complex through RHIM-dependent interactions (referred to as canonical necrosome). Necrostatin-1s (Nec-1s) blocks the cytotoxicity induced by the kinase activity of RIPK1. In TNFR1 signaling, cell death is efficiently induced only upon formation of a cytosolic cell death–inducing complex (complex II) when, for example, cellular inhibitor of apoptosis proteins (c-IAPs) are absent or depleted (IAP-antagonist treatment), TAK1 or translation is inhibited, or RIPK1 is deubiquitinated. In addition, caspases need to be inactivated or depleted, for example by pharmacological or viral inhibitors, to allow necroptosis to occur. TRIF and DAI are two other RHIM-containing adaptors that can activate RIPK3 via RHIM-dependent interactions in response to TLR3/-4 and MCMV, respectively (referred to as noncanonical necrosomes). RIPK3, upon activation by RIPK1, TRIF or DAI, phosphorylates itself and subsequently MLKL to stimulate oligomerization and translocation of MLKL to intracellular and plasma membranes. It is assumed that MLKL induces membrane rupture by binding directly to membrane phospholipids or indirectly with the help of calcium or sodium ion channels. Necrosulfonamide targets human MLKL and blocks necroptosis induction. *DAI* DNA-dependent activator of interferon regulatory factors, *DR* death receptor, *FADD* Fas-associated protein via a death domain, *IFN* interferon, *MCMV* murine cytomegalovirus, *MLKL* mixed lineage kinase domain-like, *NSA* necrosulfonamide, *LPS* lipopolysaccharide, *RIPK* receptor-interacting protein kinase, *TCR* T cell receptor, *TLR* Toll like receptor, *TNF* tumor necrosis factor, *TRIF* Toll-interleukin-1 receptor domain-containing adaptor inducing interferon-β

In this review, we will briefly describe cell death signaling downstream of TNFR1, with a focus on necrosome activation. A cytosolic cell death-inducing complex is formed upon stimulation of TNFR1 only in conditions that sensitize to cell death, for example when cellular inhibitor of apoptosis proteins (c-IAPs) are absent (IAP-antagonist treatment), TAK1 or translation is inhibited, or RIPK1 is deubiquitinated [18]. This cytosolic complex (often referred to as complex II), which is composed of at least RIPK1, the death-fold-containing proteins Fas-associated protein via death domain (FADD), CASP8, and cFLIP (Fig. 1), induces either apoptosis or necroptosis. The formation and/or activity of complex II is tightly regulated by inhibitor κB kinases (IKKs) through mechanisms that are either dependent or independent of NF-κB [27].

Necroptosis is typically initiated when caspases are insufficiently activated or their activity is blocked, for example by pharmacological or viral inhibitors. This concept was first proposed based on studies of DR signaling in fibrosarcoma cells [28, 29], and later confirmed in vivo by the rescue of the embryonal lethality of CASP8 or FADD deficiency by RIPK3 depletion [9, 14, 16]. CASP8 cleaves and inactivates RIPK1 [30], RIPK3 [31] and cylindromatosis (CYLD) [32]. This could at least partially explain the protective role of CASP8 against necroptosis [33, 34]. It is thought that CYLD is crucial for translocation of RIPK1 from receptor-bound complex I to the cytosolic death-inducing complex II by removing ubiquitin chains from RIPK1 [35, 36]. However, recently it became clear that complex II is also ubiquitylated, although the E3 ligases have not been identified yet [37–41]. While complex II is formed through interactions that depend on the death effector domain (DED) and the death domain (DD), RIPK1 and RIPK3 interact through RIP homotypic interaction motifs (RHIM) [6, 42–45]. It is assumed that this RHIM-dependent binding of RIPK1 and RIPK3 involves a conformational change that releases the RHIM domain [46]; the conformational change depends on posttranslational modifications, particularly the phosphorylation and ubiquitination status. A series of auto- and cross-phosphorylations between RIPK1 and RIPK3 result in the formation and activation of the canonical necrosome [43, 47], which appears as an amyloid-like structure of RHIM-dependent oligomerized RIPK3 [48]. The phosphorylation of human RIPK3 at Ser227 and mouse RIPK3 at Ser232 is crucial for recruitment of mixed lineage kinase domain-like (MLKL) [49–52]. Subsequent phosphorylation of MLKL at Thr357/Ser358 by human RIPK3 [50] or at Ser345/Ser347/Ser352/Thr349 by mouse RIPK3 [52] stimulates its oligomerization and translocation to intracellular and plasma membranes. The precise mechanism by which MLKL induces membrane rupture is controversial. Some reports implicate the influx of calcium or sodium through ion channels [53, 54] whereas others show direct binding to membrane phosphatidylinositol phosphates and loss of membrane integrity [55, 56].

In addition to TNF receptor signaling, other receptors induce necroptosis through RIPK1-dependent necrosome activation (Fig. 1; Table 1). These receptors include CD95L (FasL/APO-1L) [57], TRAIL (TNF-related apoptosis-inducing ligand or Apo2L) [57], TWEAK (TNF-like weak inducer of apoptosis) [58], and T cell receptor (TCR) [59]. Also, genotoxic stress [60, 61] and some anti-cancer drugs such as shikonin [62, 63] and obatoclax [64] have been shown to induce RIPK1-dependent necroptosis (Table 2). However, the assumption that many chemotherapeutics induce RIPK1/3-mediated necroptosis was recently challenged [65].

**Table 1:** Overview of experimental findings of ligand-induced necroptosis involving MLKL

Trigger	Major finding	References
TNF	Discovery of MLKL downstream of RIPK3	[50, 125]
	Quantitative phosphoproteomic analysis of RIP3-dependent protein phosphorylation	[126, 127]
	Trimerized MLKL acts at plasma membrane to allow Ca^2+^ influxvia TRPM7	[53]
	MLKL unleashes four-helical bundle to induce membrane rupture Discovery of mouse MLKL targeting compound 1	[128]
	Depletion of RIPK3 or MLKL favors RIPK1 kinase-dependent apoptosis	[92]
	RIPK1 can function as an inhibitor rather than an initiator of RIPK3-dependent necroptosis	[129]
	Hsp90 and Cdc37 are required for RIPK3 activation	[130]
	Necroptosis is preceded by nuclear translocation of RIPK1, RIPK 3 and MLKL	[131]
	Radical scavengers attenuate necrosome assembly	[132]
	Ponatinib and pazopanib inhibit necroptosis by targeting RIPK1	[133]
	Structure-guided design of potent and selective ponatinib-based hybrid inhibitors for RIPK1	[134]
	Clnk promotes necroptosis downstream of RIPK3 and upstream of MLKL	[135]
	Intracellular NAD^+^ promotes TNF-induced necroptosis in a sirtuin-dependent manner	[136]
	TRAF2 suppresses necrosome assembly	[117]
	MLKL and cofilin-1 translocation to mitochondria, Bax/Bak oligomerization, CypD and downregulation of Mcl-1 contribute to necroptosis	[137]
	Ser345 is crucial for MLKL translocation and subsequent necroptosis	[138]
LPS	RIPK3 promotes cell death and NLRP3 inflammasome activation in the absence of MLKL	[139]
	MLKL and PGAM5 upstream of NLRP3-mediated IL-1b maturation in caspase-8-deficient dendritic cells	[94]
TLR1/2,3,4,7/8 ligands	Caspase blockade induces RIP3-mediated programmed necrosis in Toll-like receptor-activated microglia	[140]
polyIC/ATP	Caspase-8 scaffolding function and MLKL regulate NLRP3 inflammasome activation downstream of TLR3	[141]
α-GalCer	Regulation of NKT cell-mediated immune responses to tumors and liver inflammation by mitochondrial PGAM5-Drp1 signaling	[142]
RIPK3 dimerization	Necroptosis is dependent on MLKL but not on DRP1	[143]
MLKL dimerization	MLKL compromises membrane integrity by binding to phosphatidylinositol phosphates	[55]
RIPK1 and -3 oligomerization	RIPK3 homo-oligomerization results in amyloid scaffold, RIPK3 autophosphorylation and MLKL docking	[144–146]
CD40L	CD40L induces necrosome-dependent necroptosis in low-grade serous carcinomas	[147]

**Table 2:** Overview of experimental findings of chemical-, toxin- or virus-induced necroptosis involving MLKL

Trigger	Major finding	References
BV6/MS275/zVADfmk	Combination of SMAC-mimetics, HDAC inhibitors and caspase inhibition induces RIPK1- and MLKL-dependent necroptosis	[148]
NPe6-PDT	Photodynamic therapy using a high dose of the photosensitizer talaporfin sodium induces RIPK1, RIPK3 and MLKL-dependent necroptosis	[149]
CCCP	Impaired oxidative phosphorylation regulates MLKL-dependent necroptosis	[150]
Cigarette smoke extract	Nex-5 and Nec-1 protect against cell death induced by cigarette smoke extract	[151]
Shikonin	RIPK1- and RIPK3-dependent necroptosis	[152]
Obatoclax	Necrosome formation at autophagosomal membranes	[64]
Staurosporine/zVADfmk	NSA blocks S/Z-induced necroptosis	[153]
Cytarabine/SMAC-mimetic	Nec-1 or NSA block TNF-mediated necroptosis	[154]
Thapsigargin tunicamycin	RIPK1/RIPK3/MLKL-mediated necroptosis in mouse fibrosarcoma cells	[155]
Edelfosine	RIPK1/RIPK3/MLKL-mediated necroptosis in human glioblastoma cells	[156]
Cisplatin	RIPK1-dependent necroptosis in squamous cell carcinoma	[157]
Doxorubicine etoposide	RIPK3-/MLKL-dependent cell death	[158]
Homoharringtonine	Sensitizes TRAIL-induced necroptosis	[159]
Acetylcholinesterase-R peptide	RIPK1/MLKL-mediated necroptosis in human granulosa cells	[160]
PMA	Neutrophil extracellular trap formation can involve RIPK1-RIPK3-MLKL signaling	[161]
	NET formation can occur independently of RIPK3 and MLKL signaling	[162]
CNOT3-deficiency	CNOT3 suppression promotes necroptosis by stabilizing mRNAs for cell death-inducing proteins	[163]
Neoalbaconol	Neoalbaconol induces cell death through necroptosis by regulating RIPK-dependent autocrine TNF	[164]
Chal-24	A JNK-mediated autophagy pathway that triggers c-IAP degradation and necroptosis for anticancer chemotherapy	[165]
Hypoxia	Glycolytic pyruvate scavenging of mitochondrial superoxide protects against necroptosis	[166]
Honokiol	Honokiol induces RIPK3- and CypD-mediated necroptosis	[167]
RNA viruses	RIPK1/RIPK3/DRP1-mediated NLRP3 activation	[168]
CMV	DAI complexes with RIPK3 to mediate virus-induced programmed necrosis	[74]
	Human CMV blocks TNF-induced necroptosis downstream of RIPK3 and MLKL	[169]
HSV-1	Mouse ICP6 triggers RIPK3/MLKL-dependent necroptosis	[170]
*Staphylococcus aureus* toxin	SA toxin triggers RIPK1/RIPK3/MLKL-dependent necroptosis	[107]
HSV-1 and -2	Human, but not mouse, HSV-1 and -2 prevent necrosome-dependent necroptosis	[171–173]
HIV	HIV-1 protease cleaves RIPK1 and RIPK2	[174]

## TRIF-dependent necroptosis

RIPK1 is the central RHIM-containing protein involved in the activation of RIPK3 during TNF-induced necroptosis, which leads to the formation of the so-called canonical necrosome complex. However, in response to some Toll-like receptors (TLRs), the RHIM-containing protein TRIF somehow activates RIPK3 independently of RIPK1 [44, 45], leading to assembly of the noncanonical necrosome complex [46]. Each member of the TLR family senses particular pathogen-associated molecular patterns [66]. When activated, TLRs recruit adaptors containing the Toll/IL-1R (TIR) domain and initiate NF-κB and IRF3/7 signaling that trigger the expression of cytokines, chemokines and interferons. All TLRs, with the exception of TLR3 and TLR4, mediate the signal through the adaptor myeloid differentiation primary-response gene 88 (MYD88). On the other hand, TLR3 and TLR4, after binding of the ligand (dsRNA and LPS, respectively), recruit the RHIM domain containing adaptor TRIF [67]. Both TLR3 and TLR4 directly induce apoptosis or, if caspase activity is compromised, necroptosis [42, 68, 69]. Several other TLRs, such as TLR2, TLR5 and TLR9, also induce cell death but through an endocrine or paracrine TNF-dependent mechanism [70]. TLR3/4-induced necroptosis is critically dependent on RHIM-mediated recruitment of TRIF to activate RIPK3 (Fig. 1) [69, 70]. Like RIPK1 and RIPK3, TRIF is a cleavage substrate of CASP8 that inhibits its ability to stimulate NF-κB-dependent cytokine expression [71]. Notably, RIPK1 seems to have a cell type specific function in TLR3/4-induced cell death. While fibroblast and endothelial cells undergo TLR3-dependent necroptosis independently of RIPK1, macrophages require RIPK1 to commit to TLR3/4-mediated necroptosis [70].

## DAI-dependent necroptosis

In addition to RIPK1 and TRIF, a third RHIM-containing protein, DAI, has been reported to activate the necrosome (Fig. 1). The DAI pathway is typically activated in response to DNA viruses and leads to inhibition of viral replication [72]. Like TLR signaling, the intracellular DNA sensor activates the NF-κB and IRF3 pathways to promote the synthesis of cytokines and interferons, which is dependent on RHIM-mediated recruitment of RIPK1 [73]. In addition, in response to DNA viruses, DAI induces necroptosis through RHIM-mediated activation of RIPK3 in the noncanonical necrosome [74]. As a virus-encoded countermeasure, the murine cytomegalovirus (CMV) M45-encoded viral inhibitor of RIP activation (vIRA) acts as a RHIM competitor and blocks necroptosis, which explains the virus’s successful replication in the host. The potency of this cell autonomous host defense pathway is demonstrated by the remarkable attenuation of M45-deficient viruses in mice. Importantly, as in RIPK3-deficient mice, mCMV lacking M45 has the same pathogenesis in DAI-deficient mice, consistent with the notion of the existence of a DAI–RIPK3 complex as the natural target of M45 [74]. M45 encodes a ribonucleotide reductase (RNR) lacking enzymatic activity. Interestingly, many RNRs from herpesviruses also encode a RHIM [75]. This suggests that viral inhibitors that target the RIPKs via the RHIM represent a common viral evasion strategy.

## The Janus faces of RIPK1

The ‘two faces’ of RIPK1 refers to its dual role. It has a cell death inhibitory role that is shown by the massive cell death observed in RIPK1-deficient models, whereas its necroptosis-inducing capacity is executed by its kinase activity. In the absence of RIPK1, massive apoptosis is observed in cells [34, 76], in postnatal death knockout mice [77], and in intestinal specific knockout mice [12, 13]. It was initially thought that this was due to the role of RIPK1 in mediating NF-κB activation, which results in the expression of survival genes such as *Flip*_*L*_ [77]. In this respect, cFLIP_L_-CASP8 heterodimers have partial enzymatic activity, leading to incomplete cleavage of CASP8 [78, 79], and this consequently prevents apoptosis. Nevertheless, it is thought that CASP8 has some local activity within complex II resulting in cleavage of RIPKs and CYLD [80], which may contribute to the anti-necroptotic role of CASP8. However, mounting evidence questions the necessity or uniqueness of the role of NF-κB activation in controlling cell death. For example, NF-κB is still activated in response to TNF stimulation in the absence of RIPK1 in cultured MEF cells [81] and in intestinal organoids [13]. TAB2-deficient mice have a functional NF-κB pathway, yet they die from massive liver apoptosis like mice deficient in p65, IKKβ, TAK1 or NEMO [82]. Moreover, the rescue of mutant RIPK1 kinase-dead knockin mice from TNF-induced shock [10, 11, 83, 84] and from the lethal TNF-induced inflammation in Sharpin mutant mice [83] also calls into question the dominance of NF-κB activation (that occurs in a RIPK1 kinase independent way). This is underscored by the recent finding that IKKα and IKKβ control RIPK1-mediated cell death independently of NF-κB activation [27].

The dual role of RIPK1 in controlling cell death is also illustrated by the perinatal death of RIPK1 knockout mice due to the aberrant activation of caspase-8 and RIPK3; mice lacking all three enzymes survived to adulthood [10, 14, 85]. Indeed, in addition to its anti-apoptotic role, RIPK1 also prevents RIPK3-driven necroptosis promoted by IFN and the TLR-adapter TRIF [14]. Since RIPK1 is reported to be essential for RIPK3 activation and subsequent necroptosis induction by TNF, the identification of settings in which RIPK1 actively suppresses RIPK3 was surprising. Moreover, conditional depletion of RIPK1 leads to apoptosis in the intestine and necroptosis in the skin [12, 13]. This dynamic interplay and interdependence of these complex II components confers a crucial host-defense function to limit pathogen spread, especially when any one of these processes is disrupted [72, 86]. This may explain why this complex interrelationship exists and why ablation of specific elements (including RIPK1, FADD, caspase-8 and cFLIP) push the system to lethality [87]. In line with this reasoning, the tissues most affected by disruption of these gene products (intestine, lung, skin, endothelium, hematopoietic cells) represent crucial barriers to infection that are constantly engaged by pathogens [88]. Depending on the tissue, cell type and developmental stage, RIPK1 can certainly either activate or inhibit cell death.

## The pleiotropic role of RIPK3

Whereas RIPK3 knockout mice are viable and fertile [5, 89], RIPK3 D161N kinase dead knockin mice die on embryonic day E10.5 due to massive levels of apoptosis in the embryo and yolk sac vasculature [11]. But this was not observed in RIPK3 D51A kinase dead knockin mice Mandal et al. [89]. The embryonic death of RIPK3 D161N kinase dead knockin mice was rescued by ablation of RIPK1 or caspase-8, indicating that RIPK3 can engage both RIPK1 and caspase-8 [11]. It remains unclear structurally why the D161N kinase-dead mutation in RIPK3 is proapoptotic, though it is likely that the kinase domain functionally “masks” the RHIM domain to prevent spurious activation [90]. In this scenario, the D161N alters the conformation of RIPK3 so that the RHIM domain is exposed for binding to RIPK1 to initiate apoptosis. This model predicts that the kinase and RHIM domains collaborate to control scaffolding of the necroptotic and apoptotic machineries. Some RIPK3 inhibitors were also found to induce apoptosis in a similar way through RHIM-dependent RIPK1 docking and subsequent FADD/CASP8-mediated apoptosis [70, 89]. Note that RIPK3 has also been reported to positively contribute to RIPK1-dependent apoptosis independently of its kinase activity but remarkably also of its RHIM domain [37]. While TNF signaling typically requires RIPK1 to activate RIPK3 in order to induce necroptosis, it has been noted that TNF can trigger RIPK3 activation even in the absence of RIPK1 if RIPK3 levels are high enough [91]. In the absence of RIPK1 and the presence of elevated levels of RIPK3, TNF can activate RIPK3 to induce cell death by both a caspase-8-dependent mechanism and a caspase-independent mechanism [37, 91]. Finally, similar to depletion of RIPK1 [34, 76], blocking TNF-induced necroptosis by suppressing RIPK3 or MLKL toggles the cell death response to apoptosis, albeit with different kinetics [92]. Collectively, these studies indicate that precise control of the complex II machinery is necessary to prevent a lethal imbalance of necroptotic or apoptotic pathways.

## Concluding remarks

There has been a revival of interest in the close interconnection between cell death and inflammation originally recognized by Virchow. This interconnection is emphasized by some recent findings that classical cell death inducers such as caspase-8 and RIPK3 seem to act also upstream of inflammasome activation in a cell autonomous way [93–98]. However, the precise mechanisms of this interaction are unclear. RIPK1 as well as RIPK3 and other cytosolic TNFR complex II components have been implicated in regulating cell death and inflammation, though if these functions could be uncoupled is not clear. In addition, the potential signal transduction interplay between parenchymal cell necrosis and some forms of necrosis that occur in immune cells, such as pyroptosis and netosis, remains unknown. Considering the central role of RHIM domains in controlling the cell death induced by several stimuli, small molecules that disrupt RHIM signaling might also be therapeutically useful.

The number of genetic (Table 3) and pharmacological studies (Table 4) demonstrating an important role for RIPK1, RIPK3 or MLKL in murine experimental disease models is still increasing, highlighting the therapeutic potential of these necrosome members. In addition, the expression and activation of RIPK1, RIPK3 and MLKL is being increasingly explored in biopsies of patients with particular pathologies driven by cell death and inflammation (Table 5). The availability of new phospho-specific antibodies, pharmacologic inhibitors and transgenic models will allow us to document further the role of necroptosis in degenerative, inflammatory and infectious diseases. It is noteworthy that therapeutic targeting of only necroptosis might be insufficient in some complex pathologies, as exemplified by the additive protective effect of targeting different types of regulated necrosis [99–101]. This observed redundancy of necrosome proteins and interplay between different modalities of necrotic cell death in vivo is an intriguing topic for further research and will generate further insight into how the targeting of these molecules in some cases looks very effective.

**Table 3:** Overview of genetic necroptosis studies in experimental mouse pathologies

Genetic defect	Major finding	References
RIPK3^KO^	Protects against pancreatitis	[6, 42]
	Sensitizes to vaccinia virus infection	[43]
	Protects against photoreceptor loss (+Nec-1)	[102]
	Protects against TNF-induced SIRS	[103]
	Partially protects from macrophage death and enhances bacterial control upon Salmonella infection	[104]
	Rescues the mid-gestation defect of cIap2/1-deficient embryos	[105]
	Protects against intracerebroventricular injection of TNF in mouse hippocampus	[106]
	Improves clearance of *Staphylococcus aureus* (+Nec-1s)	[107]
	Diminishes cutaneous wound healing	[108]
	Protects against ethanol-induced liver injury	[109]
	Protects from macrophage necroptosis in atherosclerosis development	[110, 111]
	Rescues mild systemic inflammatory disease in macrophage-specific CASP8 knockouts	[112]
	Protects against cone cell necrosis in a mouse model of inherited degeneration	[113]
	Protects against dsRNA-induced retinal degeneration	[114]
	Protects against myocardial infarction	[115]
	Protects against non-alcoholic steatohepatitis	[116]
	Partial rescue of TRAF-2-deficient mice	[117]
	Protects against Gaucher’s disease	[118]
	Protects against Ppm1b-mediated sensitization to TNF-induced SIRS	[119]
	Protects RIPK1E-KO from skin inflammation	[12]
MLKL^KO^	Deficiency of RIPK3 or MLKL does not protect against shock induced by cecal ligation and puncture MLKL deficiency protects against pancreatitis	[7]
	Nec-1 treatment and RIPK3 or MLKL deficiency protect against cisplatin-induced acute kidney injury	[120]
	Acetaminophen-induced liver toxicity occurs through RIPK1 but independently of RIPK3 and MLKL	[121, 122]
	No difference in diabetes onset by RIPK3- or MLKL-deficiency	[123]
	RIPK3 or MLKL deficiency partially protects from inflammation induced by sharpin deficiency	[124]
RIPK1^IEC−KO^	RIPK1 protects the intestinal epithelium against apoptosis	[12, 13]
RIPK1^E-KO^	RIPK1 partially protects the skin from necroptosis	[12]
RIPK3^K51A^	Viable and fertile	[89]
RIPK3^D161N^	Embryonic lethality rescued by Casp8-deficiency	[11]
RIPK1^K45A^	Protect Sharpin-deficient mice from inflammation	[83]
RIPK1^D138N^	Protects against TNF-induced shock	[11, 84]
	Does not protect against necroptosis of FADD-deficient IEC cells	[12]
RIPK1/3/CASP8^KO^	Rescue Ripk1^−/−^ neonatal lethality by Ripk3 and Casp8 depletion	[14, 85] [10]
RIPK3/CypD^KO^	Protects against renal ischemia reperfusion injury	[100]

**Table 4:** Overview of pharmacologic targeting of necroptosis in experimental rodent pathologies

Pharmacologic agent	Major finding	References
Nec-1	Alleviates ischemic brain injury	[47]
	Is cardioprotective	[175]
	Improves outcome after controlled cortical impact	[176]
	Alleviates retinal ischemia reperfusion injury	[177]
	Alleviates spinal cord injury pathology in rats	[178]
	Alleviates injury in subtotal nephrectomised rats	[179]
Nec-1s	Nec-1s treatment reduces disease severity in experimental autoimmune encephalitis model	[180]
NSA	Nec-1 or NSA block death in models of both sporadic and familial ALS (ex vivo)	[181]
Dabrafenib	Targets RIPK3 and alleviates acetaminophen-induced liver injury	[182]
	Upregulated RIPK3 expression potentiates MLKL phosphorylation-mediated programmed necrosis in toxic epidermal necrolysis	[183]
Nec-1, SanglifehrinA, 16-86	Protects from renal ischemia reperfusion injury	[99]
Nec-1, cyclosporine A, 3-AB	Protects from remote lung injury after receiving ischemic renal allografts in rats	[101]

**Table 5:** Overview of studies linking necroptosis to human pathologies

Disease	Major finding	References
Ovarian cancer	Low MLKL expression associated with poor prognosis	[184]
Inflammatory bowel disease	High RIPK3 and MLKL expression	[185]
Drug-induced liver injury	Phosphorylated MLKL in biopsies	[56]
Chronic obstructive pulmonary disease	Increased levels of RIPK3 in lung epithelial cells	[151]
Multiple sclerosis	Increased levels of RIPK1, RIPK3 and necrosome formation in lesions	[180]
Breast cancer	Weak RIPK3 expression	[158]
Melanoma	Melanoma cell lines lack RIPK3 expression, whereas primary melanocytes strongly express RIPK3	[186]
HIV	Dysfunctional HIV-specific CD8+ T cell proliferation is associated with increased caspase-8 activity and mediated by necroptosis	[187]
Leukemia	RIPK3 is downregulated	[188]

## Abbreviations

cIAP: Cellular inhibitor of apoptosis protein
cFLIP: Cellular FLICE-like inhibitory protein
CypD: Cyclophilin D
CYLD: Cylindromatosis
DAI: DNA-dependent activator of interferon regulatory factors
DR: Death receptor
FADD: Fas-associated protein via a death domain
FasL: Fas
MLKL: Mixed lineage kinase domain-like
Nec-1: Necrostatin-1
NLR: NOD-like receptor
NLRP3: NOD-like receptor family pyrin domain-containing protein 3
PAMP: Pathogen-associated molecular patterns
PARP1: Poly(ADP-ribose) polymerase 1
RHIM: RIP homotypic interaction motif
RIG-I: Retinoic acid-inducible gene-I
RIPK: Receptor-interacting protein kinase
ROS: Reactive oxygen species
TAK1: Transforming growth factor-β-activated kinase 1
TLR: Toll-like receptor
TNFR: Tumor necrosis factor receptor
TRADD: TNFR-associated death domain protein
TRAF2: TNFR-associated factor 2
TRAIL-R: TNF-related apoptosis-inducing ligand receptor
TRIF: Toll-interleukin-1 receptor domain-containing adaptor inducing interferon-β
TWEAKR: TNF-like weak inducer of apoptosis receptor
VV: Vaccinia virus
